# SYK Is Associated With Malignant Phenotype and Immune Checkpoints in Diffuse Glioma

**DOI:** 10.3389/fgene.2022.899883

**Published:** 2022-07-15

**Authors:** Quanwei Zhou, Min Wei, Wenyue Shen, Sheng Huang, Jianfeng Fan, He Huang

**Affiliations:** ^1^ Department of Neurosurgery, Xiangya Hospital, Central South University, Changsha, China; ^2^ National Clinical Research Center for Geriatric Disorders, Xiangya Hospital, Central South University, Changsha, China; ^3^ Department of Neurology, Xiangya Hospital, Central South University, Changsha, China

**Keywords:** syk, diffuse glioma, immune infiltration, tumor microenvironment, immune checkpoint molecules, prognosis

## Abstract

**Background:** Diffuse glioma, the most common intracranial malignant tumor, is characterized by immunosuppression. The prognostic significance and potential therapeutic value of SYK remain obscure. Here, we explored the performance of SYK in predicting patient outcomes and as a therapeutic target.

**Methods:** The mRNA expression and clinical data for pancancer and normal tissues and more than 2,000 glioma samples were collected from public databases. The expression level of SYK was evaluated by qPCR and IHC. The prognostic value of SYK was assessed using the Kaplan–Meier curves and univariate and multivariate Cox regression analyses. A sequence of immune and stromal infiltration analyses was calculated based on the ESTIMATE algorithm, ssGSEA algorithm, TIMER, and single-cell analysis. The SYK-related subtypes were identified *via* a Consensus Cluster Plus analysis.

**Results:** SYK was significantly differentially expressed in multiple tumors and normal tissues. Importantly, high-expression SYK was enriched in malignant phenotypes of diffuse gliomas, which was further validated by qPCR and IHC. Survival analysis uncovered that SYK was an independently unfavorable prognostic marker in diffuse glioma. Functional enrichment analysis and immune and stromal infiltration analyses showed that SYK was involved in shaping the immunosuppressive microenvironment of diffuse glioma. Additionally, SYK expression was closely associated with some immune checkpoint molecules and M2 macrophage infiltration, which was validated by IHC and single-cell analysis. Diffuse glioma with Sub1 exhibited a worse prognosis, immunosuppressive microenvironment, and higher expression of immune checkpoint genes.

**Conclusion:** SYK is involved in shaping the immunosuppressive microenvironment and served as a promising prognosis biomarker and immunotherapeutic target for diffuse glioma.

## Introduction

Diffuse glioma, the most common primary malignant tumor in the brain (GLASS Consortium, 2018), is characterized by unclear molecular classifications and heterogeneous clinical behavior (Wang et al., 2019). The fourth edition of the World Health Organization (WHO) classification of tumors of the central nervous system (CNS) classified diffuse glioma into pathological grade II, grade III, and grade IV glioma based on its history (Louis et al., 2007). However, the fifth edition of the WHO Classification of Tumors of the Central Nervous System (CNS) integrates aberrant molecules into the WHO classification (Louis et al., 2021), which enables clinicians to have a better understanding of the prognosis and optimal therapy for patients with specific CNS tumors (Wen and Packer, 2021). Patients with low-grade diffuse gliomas may survive for multiple years, while those with grade WHO IV had a median survival of approximately 14 months (Claes et al., 2007). Diffuse glioma exhibits highly aggressive behavior and high rates of mortality and recurrence (Gritsenko et al., 2020) despite multimodal treatments including surgical resection, chemotherapy, and radiation therapy (RT) (Stupp et al., 2009). Therefore, other treatment modalities are worth exploring.

Currently, immunotherapy is becoming a pillar of modern cancer treatment (Li et al., 2019a), and many patients with advanced tumors have benefited from different immunotherapeutic approaches (Li et al., 2019b). For example, patients with melanoma and non-small cell lung are treated with immune checkpoint inhibitors, such as those targeting PD-1/PD-L1 and CTLA-4, and some have achieved complete remissions (Antonia et al., 2016; Escors et al., 2018). Several preclinical studies of glioma have demonstrated the efficacy of multiple immune checkpoint inhibitors (Cockle et al., 2016; de Groot et al., 2020). However, some glioma patients do not respond to currently available immunotherapy (Huang et al., 2017). The potential mechanisms of its therapeutic resistance remain unclear. Diffuse glioma is characterized by high immunosuppression and heterogeneity of its tumor microenvironment, which is considered to be a key regulator of the malignant progression of primary tumors (Quail and Joyce, 2017; Friebel et al., 2020). It is necessary to identify novel additional therapeutic targets that alone or in combination with other cancer immunotherapies could reshape the tumor microenvironment.

Spleen tyrosine kinase (SYK) is a non-receptor tyrosine kinase pivotal to signaling through multi-level immune recognition receptors (Mócsai et al., 2010). SYK possesses controversial pro-tumor and anti-tumor properties in hematopoietic and other cell types (Efremov and Laurenti, 2011; Tan et al., 2013; Krisenko and Geahlen, 2015). Previous studies also demonstrated a novel Mincle/SYK/NF-κB signaling pathway in which the pattern recognition receptor Mincle was highly expressed in tumor-associated macrophages, thus accelerating M2 polarization and tumor progression (Li et al., 2020). The previous literature reported that SYK is responsible for immune cell infiltration into the tumor microenvironment (Moncayo et al., 2018; Yan et al., 2021). However, the relationship between SYK expression and immune checkpoint molecules has not been reported or validated comprehensively in diffuse glioma.

In this study, we found SYK was significantly over-expressed in multiple tumors. Importantly, high SYK expression was enriched in the malignant phenotype in the four cohorts, which was further validated by qPCR and IHC, and indicated an unfavorable prognosis for diffuse glioma. We also found that high-expression SYK helped to reshape the immunosuppressed glioma microenvironment. We performed IHC to further validate the correlation between SYK expression and the expression level of CD163 and PD-L1.

## Materials and Methods

### Data Extraction and Data Processing

The expression data of SYK in 33 tumor tissues and 21 tumor cell lines from the UCSC Xena platform (https://xena.ucsc.edu/), the Genotype-Tissue Expression (GTEx) portal (https://gtexport.org/home/), and the Cancer Cell Line Encyclopedia (CCLE) database (https://portals.broadinstitute.org/ccle/about) were accessed. We integrated the mRNA expression of SYK from the GTEx database into the TCGA dataset, and the expression data were normalized by log2 (expression values + 1) conversion.

In addition, the mRNA expression and clinical data of patients with diffuse gliomas were downloaded from four public datasets, including the Chinese Glioma Genome Atlas (CGGA) dataset (*n* = 693) (http://www.cgga.org.cn), TCGA dataset (*n* = 703), GSE16011 database (*n* = 276), and the Rembrandt database (*n* = 471) (https://www.ncbi.nlm.nih.gov/geo). In the TCGA cohort, a total of 677 samples were screened from 703 cases by excluding repeated samples.

### The Human Specimens

A total of 175 diffuse glioma tissues, 37 paired tumors, and peritumoral tissues, and 22 normal brain tissues from traumatic decompression patients, were collected from Xiangya Hospital, Central South University. Informed consent was obtained from all patients, and this study was ethically approved by the ethics committee of Xiangya Hospital, Central South University.

### RNA Extraction and Real-Time qPCR

We extracted total RNA using TRIzol (Life Technologies) from fresh tissues and synthesized complementary DNA from total RNA using a Reverse Transcription Kit (Thermo Fisher Scientific). Real-time qPCR was performed with ChamQ SYBR qPCR Master Mix (Vazyme). The relative expression of different sets of genes was quantified relative to ACTB mRNA using the 2^–ΔΔCt^ method. The primer sequences for qPCR used were listed as follows: SYK forward primer 5′-GCC​ATC​GGT​TCA​GTT​CA-3′, SYK reverse primer 5′-CAA​TCC​CCG​AGT​AAG​CAT-3′; ACTB forward primer 5′-ACA​GAG​CCT​CGC​CTT​TGC​CGA​T-3′, ACTB reverse primer 5′- CTT​GCA​CAT​GCC​GGA​GCC​GTT-3′.

### Tissue Microarray and Immunohistochemistry

A Tissue Microarray (TMA) containing 78 diffuse gliomas and six normal samples from Xiangya Hospital, Central South University, was constructed. Immunohistochemistry (IHC) was performed using a primary antibody against PD-L1 (Cell Signaling Technology Pathways, United States), CD163 (Proteintech, China), and SYK (Proteintech, China). The immunostaining results were evaluated by two independent pathologists based on the intensity and percentage of membranous or nuclear reactivity, as reported in the previous literature (Zhou et al., 2016). In summary, the target protein was mainly located in the nucleus and cytoplasm, and positive staining showed brownish-yellow or tan colors. Four different fields were randomly selected under high magnification (200×), and the total number of cells and the number of positive cells were counted. The staining was scored according to the following criteria: 0 if no cells were stained, 2 if 0%–25%, 2 if 25%–50%, 3 if 50%–75%, and 4 if more than 75% of the cells were stained. We scored the staining intensity as 0 for negative, 1 for weak, 2 for moderate, and 3 for strong. The total score was obtained by multiplying the percentage score by the intensity score. Two independent pathologists who were blinded to the source of the slides examined and scored each sample.

### Survival Analysis

Samples with more than 30 days of overall survival (OS), disease-specific survival (DSS), and progression-free interval (PFI) were enrolled in the four datasets. The correlations between SYK expression and patient prognosis were assessed by the Kaplan–Meier curves in the four cohorts. The log-rank test was performed to assess the significance of SYK expression and clinical outcomes using the survival package in R. Univariate and multivariate Cox regression analyses were performed to estimate the independent prognostic factors, including age, gender, grade, SYK expression, and IDH status, in the TCGA cohort. *p* < 0.05 was considered statistically significant.

### The Differential Analysis and Functional Enrichment Analysis

Based on the median expression value of SYK in the four cohorts, all patients were divided into high-expression and low-expression groups. The differentially expressed genes between the two groups were identified using the limma package in R software (version 3.6.0), with cutoff criteria of adjusted *p* < 0.05 and absolute log_2_ (fold change) > 1 (Ritchie et al., 2015) in the TCGA cohort. Kyoto Encyclopedia of Genes and Genomes (KEGG) analysis and Gene Ontology (GO) analysis of differentially expressed genes were performed using the package clusterProfiler (Yu et al., 2012). The GO categories included cellular components (CC), biological processes (BP), and molecular function (MF). In addition, gene set enrichment analysis (GSEA) was performed to investigate the underlying signaling pathways of SYK with GSEA software (vision 3.0), which was downloaded from the Broad Institute (http://www.broadinstitute.org/gsea). We obtained normalized enrichment scores and the *p* value of each biological pathway by GSEA.

### Estimation of Immune and Stromal Infiltration

Based on the gene expression profile, we applied the ESTIMATE R package to evaluate the immune and stromal scores, which can reflect the abundance of stromal cells and immune cells (Yoshihara et al., 2013). The abundance of six types of immune cells was obtained from the Tumor Immune Estimation Resource (TIMER, https://cistrome.shinyapps.io/timer/) (Li et al., 2017). The ssGSEA method was applied to evaluate the relative levels of 35 types of immune and stromal cells (Hänzelmann et al., 2013). The gene list of each immune cell type was obtained from recent publications (Bindea et al., 2013; Charoentong et al., 2017; Jia et al., 2018). In addition, we used the MCP-counter method to determine the absolute abundance of endothelial cells and fibroblasts (Becht et al., 2016).

### Single-Cell Sequencing Analysis

We downloaded the single-cell data expression matrix of the GSE84465 database from GEO (https://www.ncbi.nlm.nih.gov/geo) (Darmanis et al., 2017), and analyzed the data with the R package of Seurat (Butler et al., 2018). After normalizing the expression data, we used “RunPCA” to perform principal component analysis (PCA). Eighteen cell clusters were identified and visualized by tSNE. We also analyzed the distribution of SYK and 10 markers of B cells, CD8^+^ T cells, CD4^+^ T cells, M1 macrophages, M2 macrophages, neutrophils, and dendritic cells in the cell clusters.

### Statistical Analysis

Unpaired Student’s t-test was performed to evaluate SYK expression between normal and tumor tissues, different grades, and wild-type and mutant IDH or IDH1, whereas the Kruskal–Wallis test was used to compare different histological subtypes. Pearson’s or Spearman’s test was conducted to analyze the correlation between SYK expression and the expression levels of the other genes. All statistical analyses were performed with GraphPad Prism 7.0 or R software (www.r-project.org). *p* < 0.05, *p* < 0.01, *p* < 0.001, and *p* < 0.0001 were considered statistically significant.

## Results

### The Expression Pattern of Spleen Tyrosine Kinase in Human Normal Tissues and Across Cancers

First, we analyzed the expression level of SYK in 32 types of normal tissues from the GTEx dataset, which suggested SYK was heterogeneously expressed in different normal tissues, with lower expression of SYK in the liver and muscle tissues, and the higher expression of SYK in the spleen, blood, and thyroid tissues (Supplementary Figure S1A) (*p* < 0.05). We also found that SYK was expressed at relatively low levels in the brain tissues (Supplementary Figure S1A) (*p* < 0.05). Furthermore, we explored the expression level of SYK in 21 types of tumor cell lines from the CCLE database, which indicated SYK was universally expressed in all tumor cell lines (Supplementary Figure S1B). To further explore the differences in SYK expression between tumor and normal tissues, we obtained SYK expression data from 25 types of tumors and normal tissues from the TCGA database. We found SYK was significantly up-regulated in the 11 types of tumors, including GBM and LGG, and down-regulated in seven types of tumor tissues (KIRC, KIRP, and LUAD) relative to normal tissues (Supplementary Figure S1C) (*p* < 0.05). However, no difference in SYK expression was observed in the seven types of tumor tissues, including BRCA, KICH, LICH, and SKCM, relative to normal tissues (Supplementary Figure S1C) (*p* > 0.05). To expand the number of normal tissues, we integrated SYK mRNA expression data from the GTEx database into those with the TCGA database. SYK expression was significantly higher in tissues from 25 types of tumors and lower in two types of tumor tissues (Supplementary Figure S1D) (*p* < 0.05). In conclusion, our results indicated that SYK was heterogeneously expressed in normal tissues and universally upregulated in pancancers tissues.

### The Expression Pattern of Spleen Tyrosine Kinase in Diffuse Glioma

**FIGURE 1:**
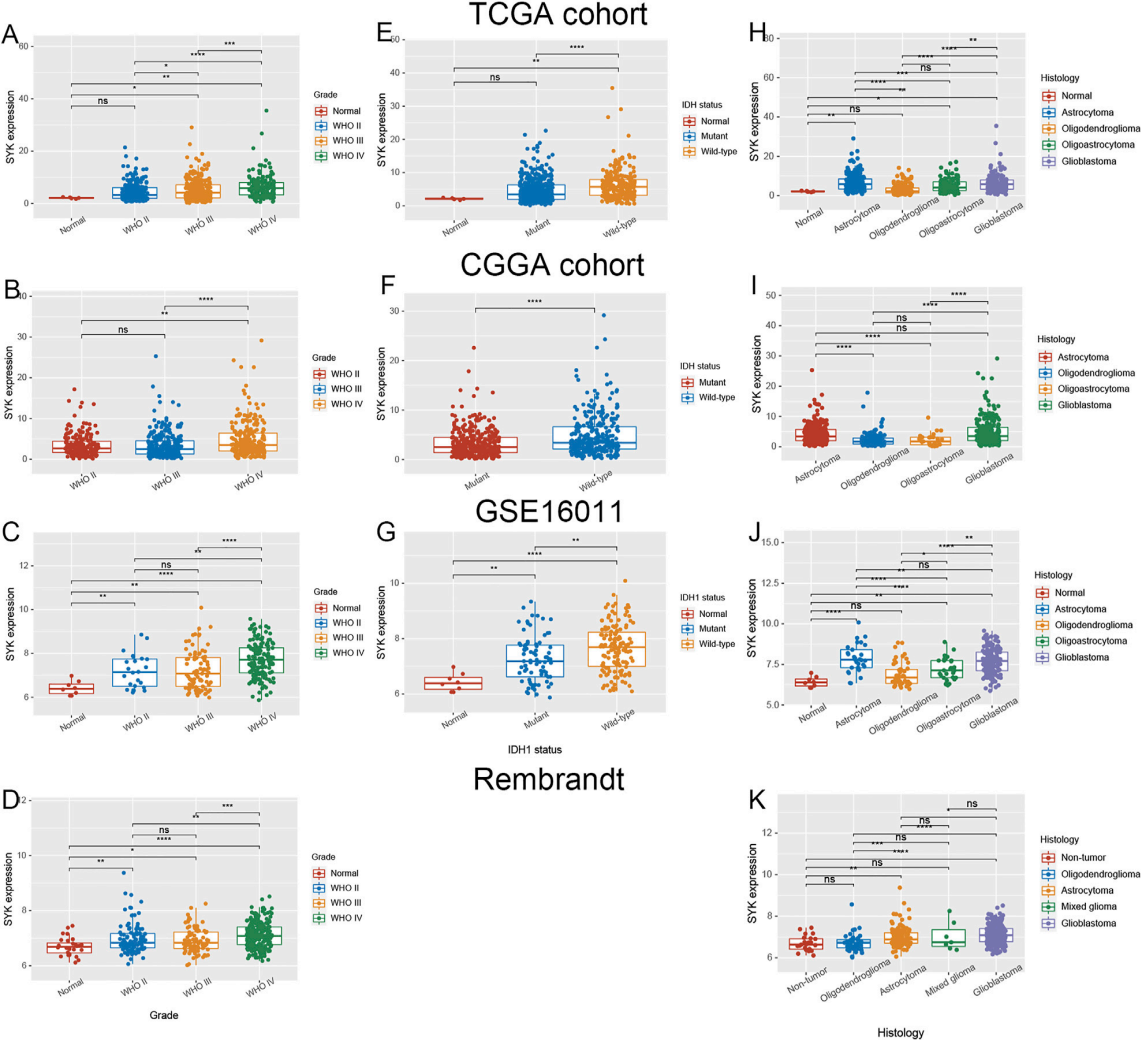
Expression pattern of SYK in diffuse gliomas. **(A–D)** The expression level of SYK in diffuse glioma with different WHO grades in the TCGA cohort **(A)**, CGGA cohort **(B)**, GSE16011 **(C)**, and Rembrandt cohort **(D)**; **(E–G)** The expression level of SYK in diffuse glioma with wild-type and mutant IDH or IDH1 in the TCGA cohort**(E)**, CGGA cohort **(F)**, and GSE16011 **(G)**; **(H–K)** The expression level of SYK in diffuse glioma with different histologies in the TCGA cohort **(H)**, CGGA cohort **(I)**, GSE16011 **(J)**, and Rembrandt cohort **(K)**. *p* < 0.05 was considered significant.

**FIGURE 2:**
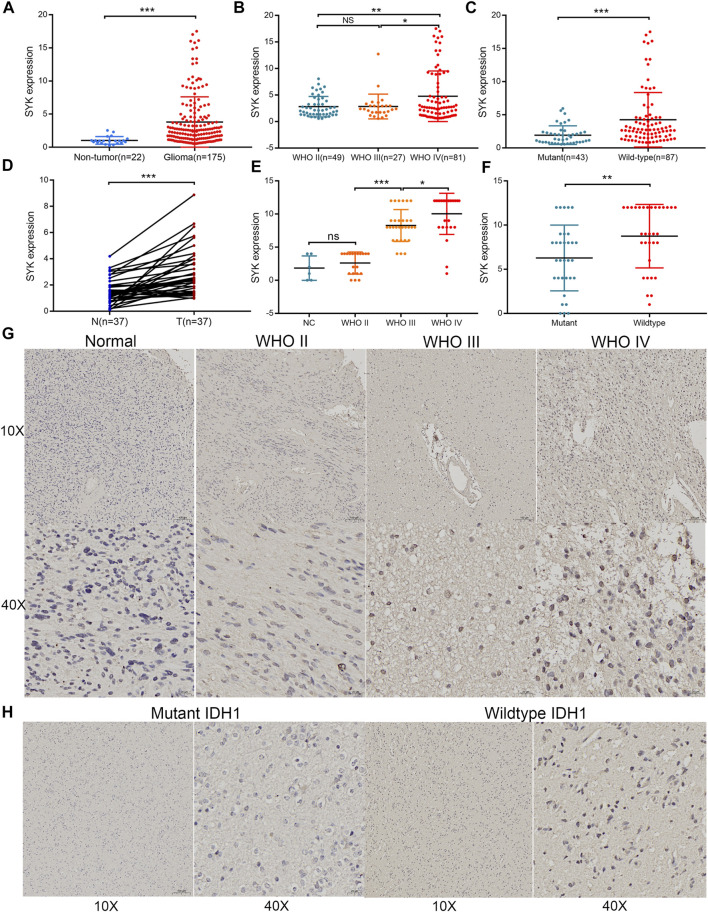
Validation of the expression pattern of SYK in diffuse gliomas analyzed by RT-qPCR and IHC. **(A)** The expression of SYK in diffuse glioma and normal brain tissues analyzed by RT-qPCR; **(B)** The expression of SYK in diffuse glioma with different WHO grades analyzed by RT-qPCR; **(C)** The expression of SYK in diffuse glioma with wildtype and mutant IDH1 analyzed by RT-qPCR; **(D)** The expression of SYK in diffuse glioma with glioma and peritumoral tissues analyzed by RT-qPCR; **(E–H)** The expression level of SYK in diffuse glioma with different WHO grades **(E,G)** and wild-type and mutant IDH1 **(F,H)** analyzed by IHC staining. *p* < 0.05 was considered significant.

At present, few studies have investigated the role of SYK in diffuse glioma, and its regulatory mechanism in diffuse glioma is not clear. Considering that SYK was significantly differentially expressed in the diffuse glioma (Supplementary Figures S1C, D) (*p* < 0.05), we further explored the correlation between SYK expression and important clinicopathological features, such as WHO grade, the mutation status of IDH or IDH1, and histology, in the four cohorts. We found SYK expression was dramatically higher in higher grade diffuse glioma than in lower grade glioma, in the TCGA cohort (*p* < 0.05, Figure 1A). Similar results were observed in the CGGA dataset, Rembrandt dataset, and GSE16011 dataset (*p* < 0.05, Figures 1B–D). In the TCGA dataset, diffuse glioma with wild-type IDH had higher SYK expression than those with mutant IDH, which was validated in the CGGA cohort and GSE16011 cohort (*p* < 0.05, Figures 1E–G). In addition, high-expression SYK was frequently enriched in the GBM in the four cohorts (*p* < 0.05, Figures 1H–K). To validate the expression pattern of SYK, we also collected 22 fresh normal and 175 tumor tissues from Xiangya Hospital, Central South University, and 37 paired tumor and peritumoral tissues. In this validation dataset, SYK was highly expressed in the glioma tissues, compared with normal tissues (Figure 2A, *p* < 0.05). We also similarly found SYK was up-regulated in glioma with WHO grade IV (Figure 2B, *p* < 0.05), and wild-type IDH glioma (Figure 2C, *p* < 0.05) by qPCR, which was consistent with the aforementioned results. In addition, SYK was significantly over-expressed in 37 pairing tumor tissues, compared with matching peritumoral tissues (Figure 2D, *p* < 0.05). We then analyzed the SYK expression in the tissue microarrays that contained 78 diffuse glioma samples and six normal samples, by immunohistochemistry (IHC) staining. We found diffuse glioma with a higher WHO grade, wild-type IDH1, and GBM exhibited higher SYK expression (*p* < 0.05, Figures 2F–H). Therefore, these observations indicated that higher-expression SYK accompanies a more malignant phenotype in diffuse glioma.

### Spleen Tyrosine Kinase Was an Unfavorable Prognostic Marker in Diffuse Gliomas

**FIGURE 3:**
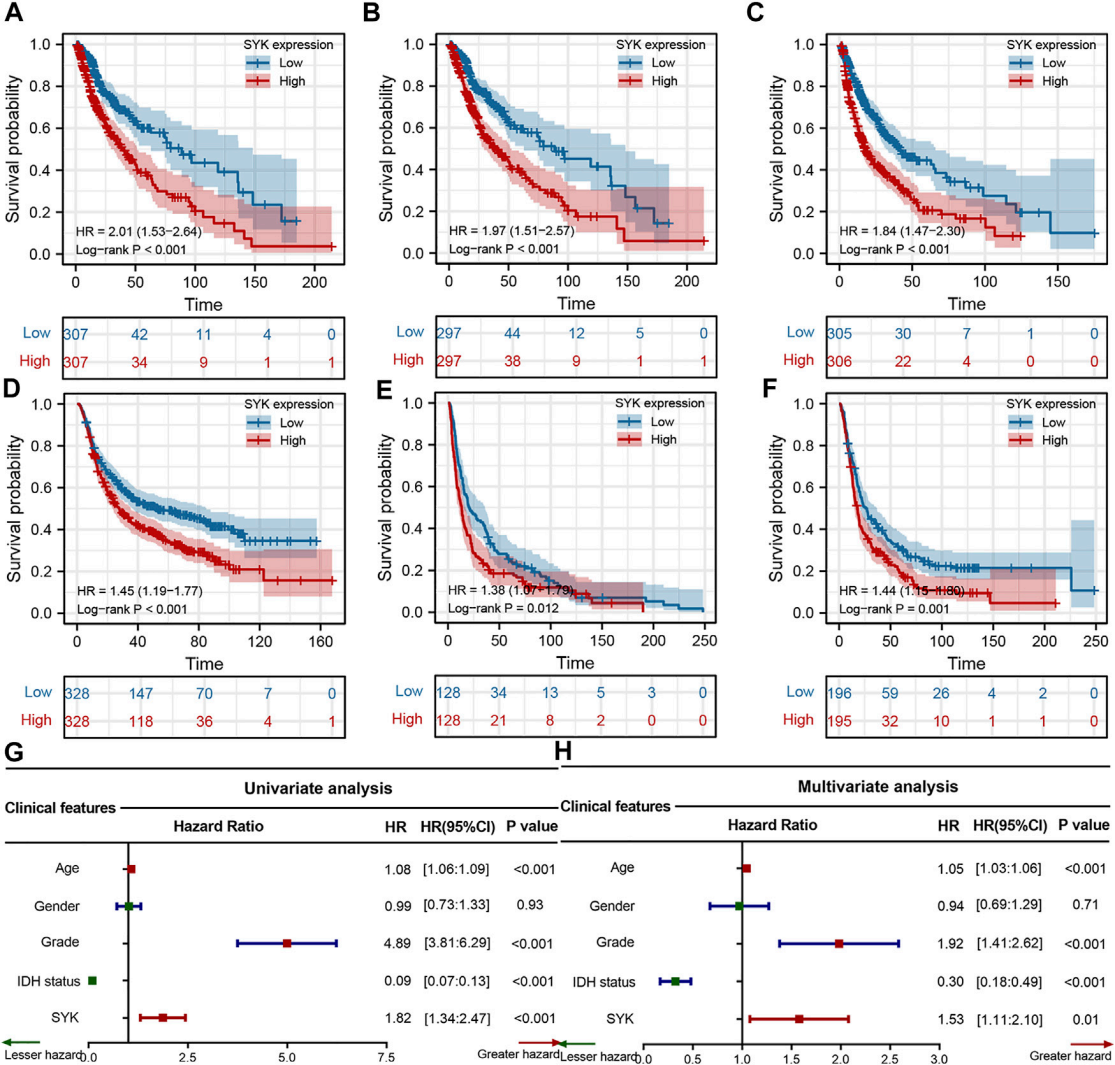
Survival analysis of SYK in the four cohorts. **(A–C)** The Kaplan–Meier curves displayed the correlation between SYK expression and OS **(A)**, DSS **(B)**, and PFI **(C)** in diffuse gliomas. **(D–F)** Kaplan–Meier curves show the correlation between SYK expression and OS of patients in the CGGA cohort **(D)**, GSE16011 cohort **(E)**, and Rembrandt cohort **(F)**. **(G–H)** The univariate and multivariate Cox regression analyses of SYK expression in the TCGA cohort. *p* values were calculated by the log-rank test, and *p* < 0.05 was considered significant.

To explore the clinical significance of SYK expression in diffuse glioma, the Kaplan–Meier curves were performed to assess the association between SYK expression and diffuse gliomas’ OS, DSS, and PFI in the TCGA cohort. The results revealed that high SYK expression predicted an unfavorable outcome in the TCGA cohort (log-rank test *p* < 0.001, Figures 3A–C). The correlation between SYK expression and OS was further validated in the other three cohorts (log-rank test *p* < 0.001, Figures 3D–F). In addition, the univariate and multivariate Cox regression analyses uncovered that SYK was an unfavorable prognostic marker, independent of age, gender, grade, and the mutation status of IDH in the TCGA cohort (the univariate Cox regression analysis, 95% CI = 2.033–3.849; the multivariate Cox regression analysis: 95% CI = 1.058–2.148) (Figures 3G, H, *p* < 0.05). Overall, these results suggested that SYK could serve as an independent predictor of a poor outcome among patients with diffuse gliomas.

### Spleen Tyrosine Kinase Was Involved in the Immunologic Processes

**FIGURE 4:**
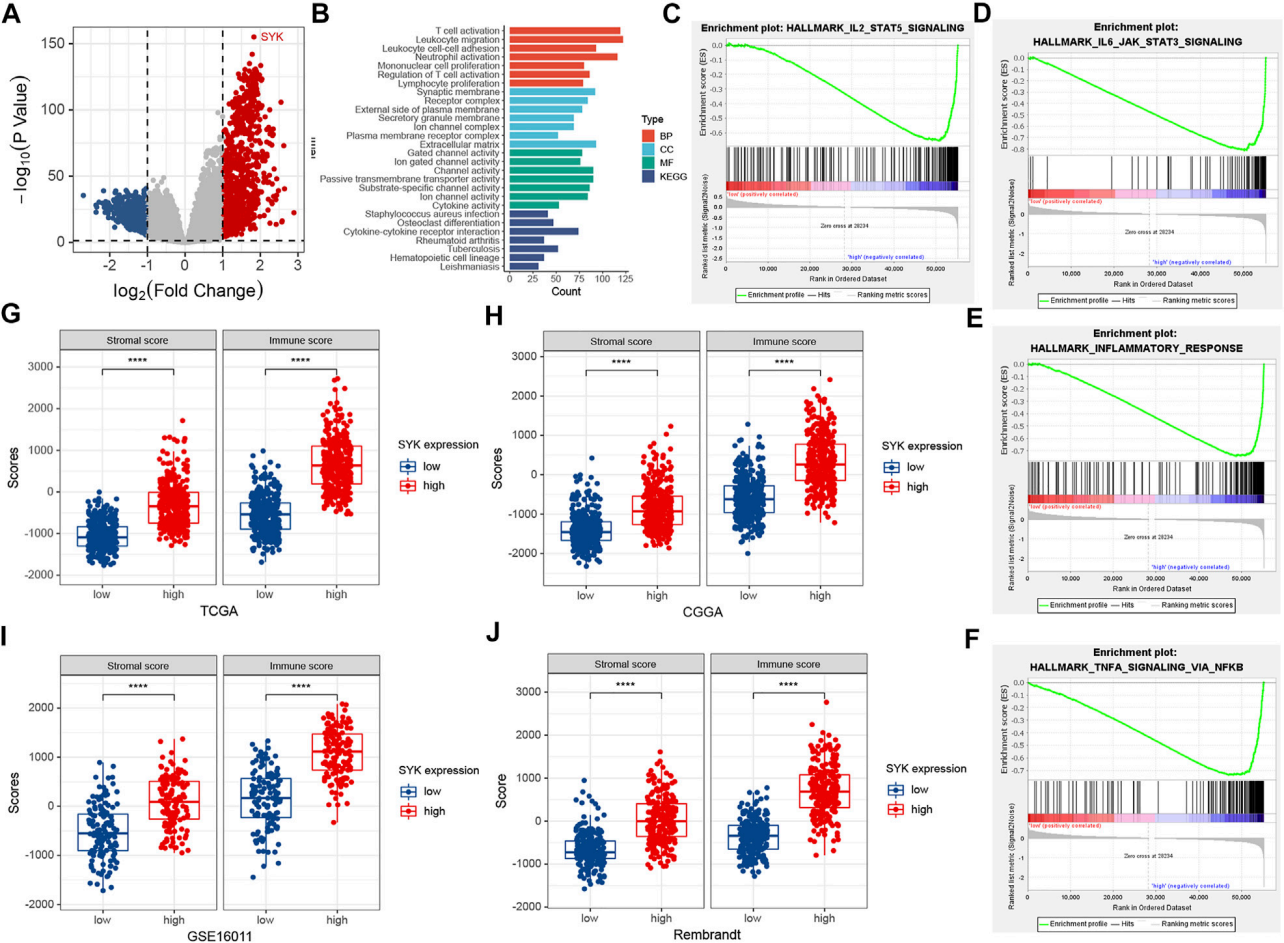
Functional enrichment analysis and the association between SYK expression and immune and stromal scores. **(A)** The volcano plot shows differentially expressed genes between the low- and high-expression SYK groups in the TCGA cohort. **(B)** The enriched pathways using GO and KEGG enrichment analyses in the TCGA cohort. **(C–F)** GSEA analysis of diffuse gliomas with the low-expression and high-expression SYK in the TCGA cohort. The thresholds with a nominal *p* < 0.05 and an FDR <25% were considered to determine the significance of the enrichment score (ES). **(G–J)** The difference in stromal and immune scores between the low-expression and high-expression SYK in the TCGA cohort **(G)**, CGGA cohort **(H)**, GSE16011 **(I)**, and Rembrandt cohort **(J)**. Differences between the two groups were compared by Student’s t-tests, and the *p* values are labeled above each boxplot with asterisks (****p < 0.0001).

To explore the potential pathways of SYK, all tumor samples were classified into low- and high-level groups based on the median value of SYK expression in the TCGA cohort. The differentially expressed genes between the two groups, including 3,684 significantly up-regulated genes and 2,910 significantly down-regulated genes, were identified with cut-off criteria of adjusted *p* < 0.05, and absolute log_2_ (fold change) > 1 in the TCGA cohort (Figure 4A). GO and KEGG enrichment analyses indicated that significantly differential genes were enriched in the immunologic processes, such as “leukocyte migration,” “T-cell activation,” “regulation of immune effector process,” and “humoral immune response” (Figure 4B). We also performed GSEA to further explore the underlying pathways of SYK. The GSEA results suggested diffuse glioma with high-level SYK group exhibited biological pathways that were involved in modulating the immune response, including “IL2/STAT5 signaling,” “inflammatory response,” “NF-kappaB signaling,” and “IL6/STAT3 signaling” in the TCGA cohort (Figures 4C–F). Then, the ESTIMATE algorithm was used to comprehensively evaluate the abundance of two important components in the complex tumor microenvironment (Yoshihara et al., 2013). We also found diffuse gliomas with high-expression SYK consistently exhibited higher immune and stromal scores, compared with those with low-expression SYK in the four cohorts (*p* < 0.05, Figures 4G–J), which indicated SYK might be involved in regulating the immune and stromal cells in the tumor microenvironment. Therefore, our findings indicate that SYK is involved in remodeling the glioma microenvironment to promote malignant progression.

### Spleen Tyrosine Kinase Shapes an Immunosuppressive Tumor Microenvironment in Diffuse Glioma

**FIGURE 5:**
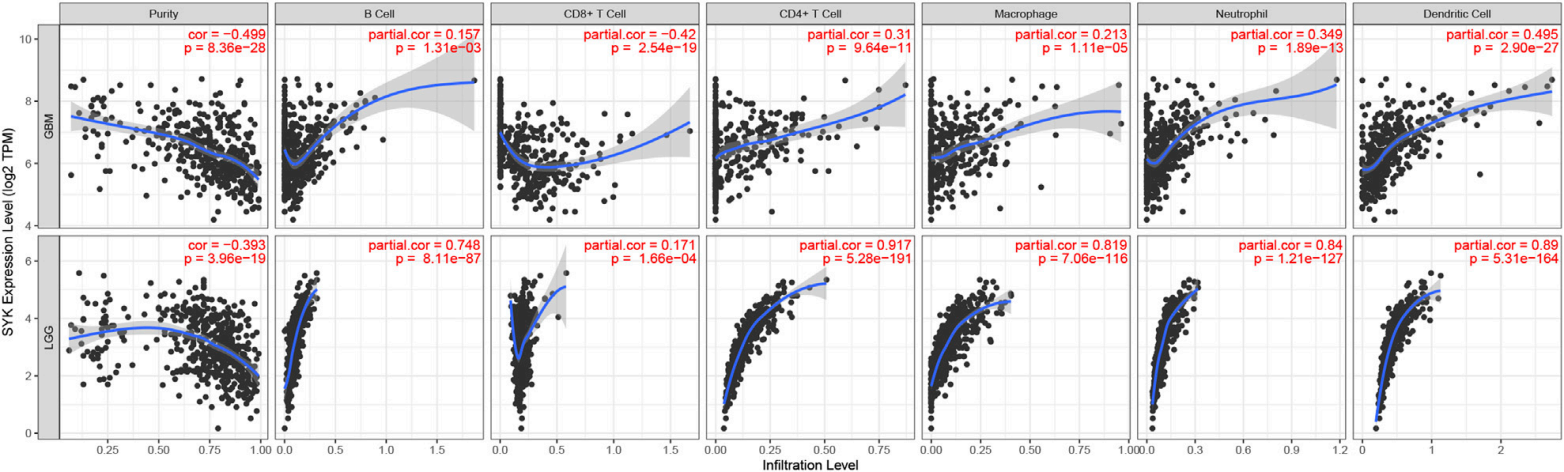
Correlation between the expression levels of SYK and immune cells in diffuse glioma based on the TIMER algorithm.

**FIGURE 6:**
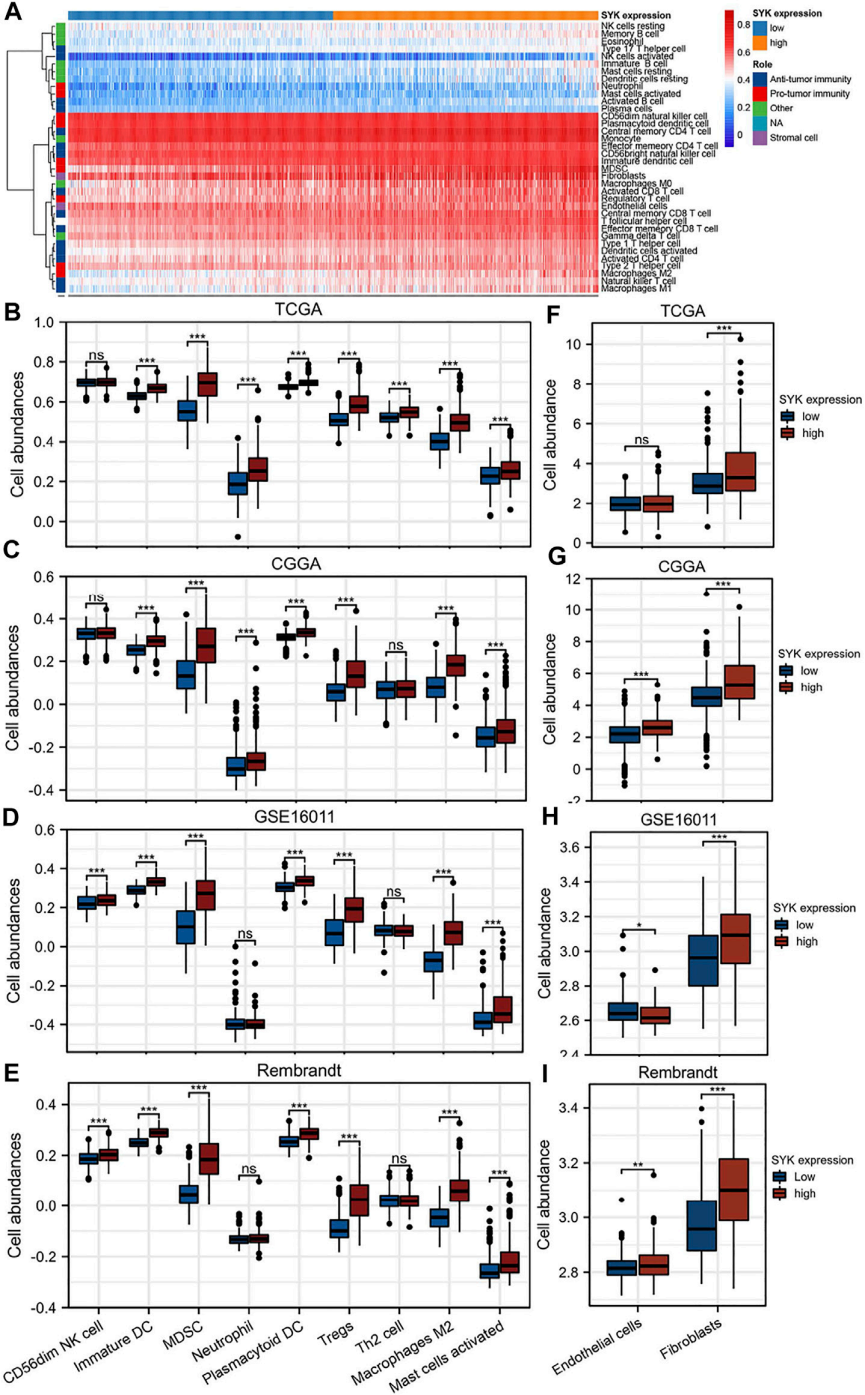
Correlation of SYK expression with immune and stromal infiltration levels in diffuse gliomas. **(A)** Heatmap showing the landscape of immune cells and stromal cells in the TCGA cohort. **(B–E)** The association between the mRNA expression of SYK and the abundance of seven types of protumor immune cells in the four cohorts. **(F–I)** The association between SYK expression and the abundance of endothelial cells and fibroblasts in the four cohorts (- no significance, *p < 0.05, **p < 0.01, and ***p < 0.001).

Considering that SYK might regulate the tumor microenvironment by immunologic biological processes, the role of SYK in the tumor microenvironment needs to be further explored in diffuse glioma. First, the correlations between SYK expression and the infiltration levels of six types of immune cells were evaluated in the GBM and LGG using TIMER website tools. We found that SYK expression was positively correlated with infiltrating levels of B cells (LGG, *r* = 0.748; GBM, *r* = 0.157), CD4+ T cells (LGG, *r* = 0.310; GBM, *r* = 0.917), macrophages (LGG, *r* = 0.819; GBM, *r* = 0.213), neutrophils (LGG, *r* = 0.840; GBM, *r* = 0.349), and dendritic cells (LGG, *r* = 0.890; GBM, *r* = 0.495) in the diffuse glioma (*p* < 0.05, Figure 5). However, SYK expression had an adverse correlation with the infiltrating levels of CD8+ T cells between LGG and GBM (*p* < 0.05, Figure 5). We then detected the distribution of different types of cells in the diffuse glioma with high- and low-levels of SYK expression. The abundance of 35 immune cell types was evaluated by the ssGSEA method in the TCGA cohort (Figure 6A). Our results uncovered diffuse glioma with high-expression SYK displayed a higher abundance of most immunosuppressive cells, except for CD56dim NK cells, in the TCGA cohort (*p* < 0.05, Figure 6B). Similar results were observed in the CGGA, GSE16011, and Rembrandt cohorts (Figures 6C–E, *p* < 0.05). However, no difference in abundance in the Th2 cells was observed in diffuse glioma with high- and low-expression SYK in the CGGA, GSE16011, and Rembrandt cohorts (Figures 6C–E, *p* > 0.05). In addition, we did not observe a difference in neutrophils in the GSE16011 and Rembrandt cohorts (Figures 6D, E, *p* > 0.05). Finally, we used the MCP-counter method to assess the absolute abundance of endothelial cells and fibroblasts in the four cohorts. We observed diffuse gliomas with high SYK expression showed a significantly higher abundance of endothelial cells and fibroblasts than those with low SYK expression (Figures 6F–I, *p* < 0.05). We did not find a significant difference in endothelial cells in the TCGA cohort (Figures 6F–I, *p* > 0.05). In conclusion, aberrant expression of SYK might be involved in shaping the immunosuppressive microenvironment in diffuse gliomas.

### Aberrant Spleen Tyrosine Kinase Expression Was Associated With M2 Macrophages

**FIGURE 7:**
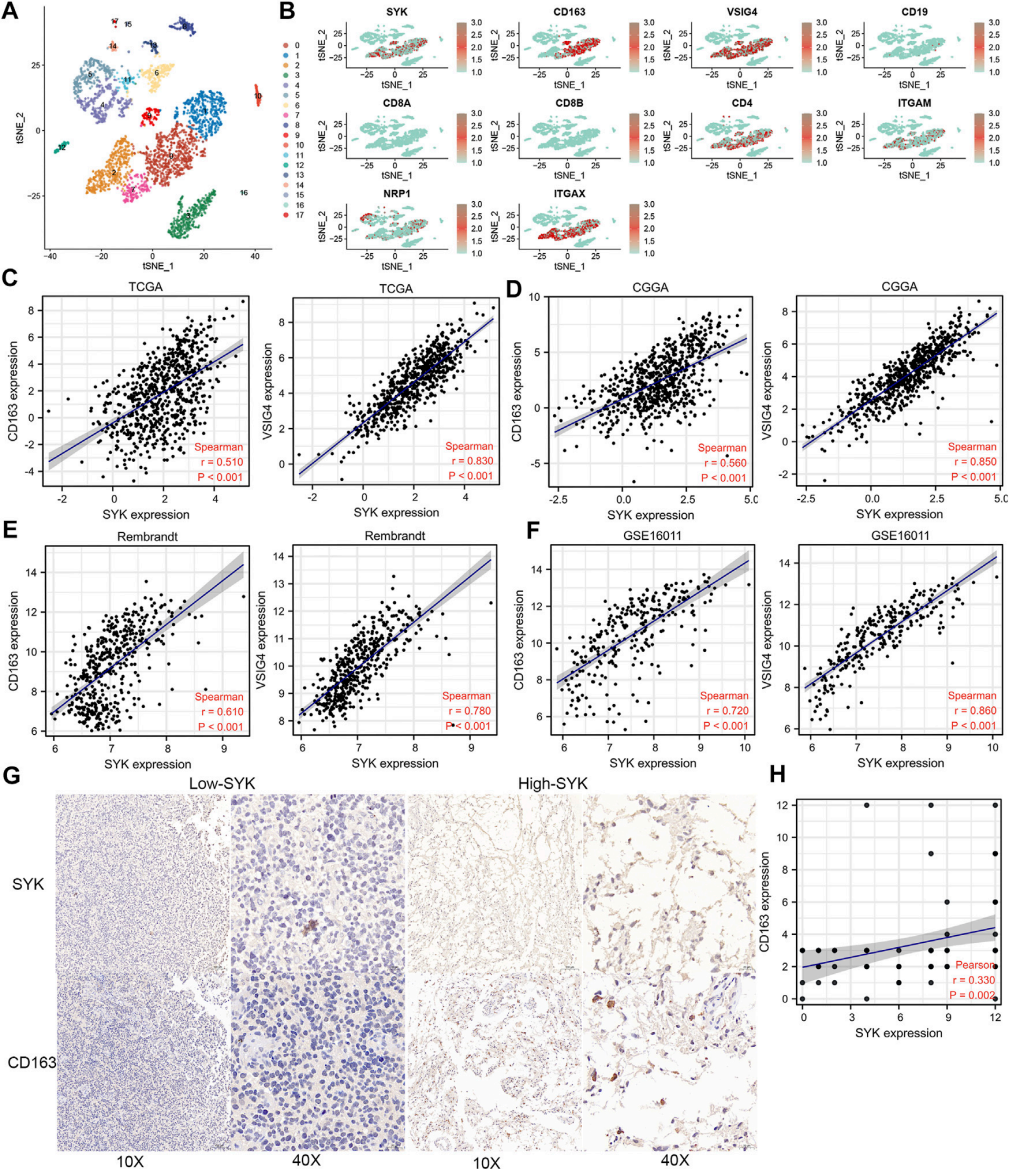
Expression pattern of SYK by single-cell sequencing analysis and the association between SYK expression and M2 macrophage infiltration. **(A,B)** SYK expression in the different cell types; **(B–F)** The correlation between SYK expression and CD163 and VSIG4 expression in the four cohorts. **(G,H)** The correlation between SYK expression and CD163 expression by IHC staining.

We performed the single-cell sequencing analysis to identify 18 cell clusters (Figure 7A), and further analyzed the distribution of SYK and 10 markers of B cells, CD8^+^ T cells, CD4^+^ T cells, M1 macrophages, M2 macrophages, neutrophils, and dendritic cells in cell clusters, such as CD163, VSIG4, CD19, CD8A, CD8B, CD4, ITGAM, NRP1, ITGAX, and NOS2. Our results uncovered that SYK and the markers of M2 macrophages, such as CD163 and VSIG4, shared a similar expression pattern, which suggested aberrant-expression SYK might recruit more M2 macrophages infiltration (Figure 7B). Consistently, we also found that SYK expression had moderate and strong correlations with the mRNA expression levels of CD163 and VSIG4 in the four cohorts (Spearman rank test, R > 0.500, *p* < 0.05, Figures 7C–F). In glioma tissue, diffuse gliomas with high-expression SYK exhibited high-expression CD163 by IHC (Figure 7G). In addition, a positive correlation between the expression of SYK and CD163 was observed (R = 0.330, *p* < 0.05, Figure 7H). These results indicated aberrant-expression SYK was associated with M2 macrophage infiltration in diffuse glioma.

### Correlations Between Spleen Tyrosine Kinase and Tumor Mutation Burden (TMB), Immune Checkpoints, and Immunogenic Cell Death

**FIGURE 8:**
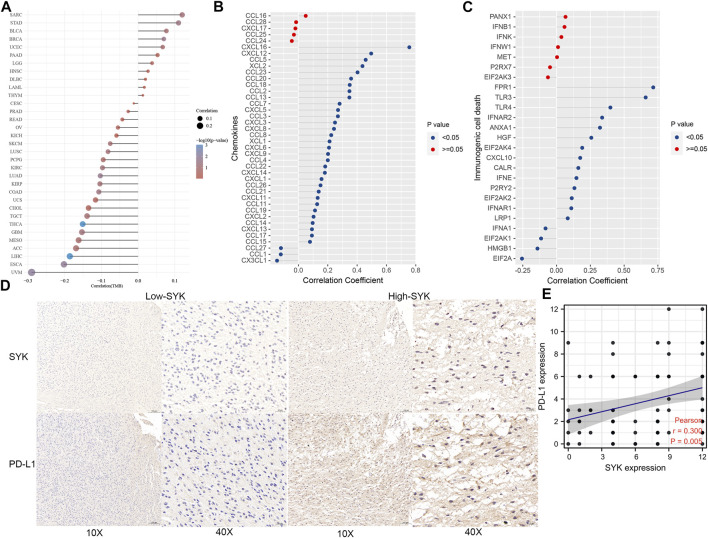
Correlation between SYK expression and TMB, chemokines and immunogenic cell death modulator-related genes, and PD-L1. **(A)** The correlation between SYK expression and TMB in pan-cancer. **(B,C)** The correlation between SYK expression and the expression of chemokines **(B)** and immunogenic cell death modulator **(C)**-related genes in diffuse glioma. **(D,E)** The correlation between SYK expression and PD-L1 expression by IHC staining.

To explore the efficacy of SYK as a synergistic partner of immune checkpoints, we first analyzed the correlation between SYK expression and the expression of immune checkpoints and immunogenic cell death in 33 types of tumors. The results showed that the expression of SYK was positively related to the expression of immune checkpoint molecules and immunogenic cell death, such as CD80, CD40, PD-L1, PD1, and PD-L2, in multiple tumors, including LGG and GBM (*p* < 0.05, Supplementary Figure S2). TMB serves as an important indicator of the efficacy of immunotherapy (Strickler et al., 2021). Consistent with the immune checkpoints, the mRNA levels of SYK were positively associated with tumor mutation burden (TMB) in multiple tumors, including GBM and MESO (*p* < 0.05, Figure 8A). Subsequently, we further analyzed the correlation between SYK expression and that of 37 immune checkpoint molecules and the corresponding ligands in the diffuse gliomas. We found SYK expression was tightly associated with some of the checkpoint molecules, such as CD276 (CGGA, *r* = 0.376; GSE16011, *r* = 0.354; Rembrandt, *r* = 0.369; and TCGA, *r* = 0.378), CD40 (CGGA, *r* = 0.581; GSE16011, *r* = 0.460; Rembrandt, *r* = 0.431; and TCGA, *r* = 0.474), CD44 (CGGA, *r* = 0.508; GSE16011, *r* = 0.632; Rembrandt, *r* = 0.599; and TCGA, *r* = 0.498), CD80 (CGGA, *r* = 0.571; GSE16011, *r* = 0.205; Rembrandt, *r* = 0.233; and TCGA, *r* = 0.489), CD86 (CGGA, *r* = 0.775; GSE16011, *r* = 0.752; Rembrandt, *r* = 0.734; and TCGA, *r* = 0.767), LAIR1 (CGGA, *r* = 0.800; GSE16011, *r* = 0.781; Rembrandt, *r* = 0.786; and TCGA, *r* = 0.758), and PD-L2 (CGGA, *r* = 0.621; GSE16011, *r* = 0.296; Rembrandt, *r* = 0.169; and TCGA, *r* = 0.565) (Table 1, Pearson test, *p* < 0.05). Although no correlation was found between SYK expression and PD-L1 expression in the GSE16011 and Rembrandt cohorts (Table 1, Pearson test, *p* > 0.05), a moderate positive correlation was shown in the TCGA (*r* = 0.369) and CGGA cohorts (*r* = 0.461) (Table 1, Pearson test, *p* < 0.05). Paradoxically, the expression of SYK was positively correlated with PDCD1 in the CGGA (*r* = 0.108) and TCGA datasets (*r* = 0.424) but negatively correlated in GSE16011 (*r* = −0.292) and Rembrandt cohorts (*r* = −0.118) (Table 1, Pearson test, *p* < 0.05). In addition, we further explored the association between SYK expression and some chemokines and immunogenic cell death (ICD) modulator-related genes and found the expression of SYK was closely correlated with the expression of most chemokines and ICD modulator-related genes in diffuse glioma (Pearson test, *p* < 0.05, Figures 8B,C). Finally, we explored PD-L1 expression using glioma tissue microarrays as mentioned earlier (Figure 2). A positive correlation was observed between SYK expression and the expression of PD-L1 (Pearson test, *r* = 0.300, *p* < 0.05, Figures 8D,E). Our findings suggest that blocking SYK might have improved synergistic effects with immune checkpoint inhibitors.

**TABLE 1:** Correlation analysis between SYK expression and the mRNA expression of immune checkpoint markers in diffuse gliomas.

Gene	The TCGA cohort		The CCGA cohort		The rembrandt cohort		GSE16011	
	Pearson r	*p* value	Pearson r	*p* value	Pearson r	*p* value	Pearson r	*p* value
BTLA	0.457	<0.001	0.345	<0.001	0.310	<0.001	0.138	0.024
CD160	0.136	<0.001	0.218	<0.001	−0.026	0.604	−0.077	0.208
CD200	−0.251	<0.001	−0.059	0.120	−0.255	<0.001	−0.324	<0.001
CD200R1	0.530	<0.001	0.528	<0.001	0.201	<0.001	0.211	<0.001
CD244	0.459	<0.001	0.463	<0.001	0.098	0.050	0.072	0.241
CD27	0.340	<0.001	0.306	<0.001	0.393	<0.001	0.133	0.030
CD274	0.300	<0.001	0.484	<0.001	0.045	0.366	0.089	0.146
CD276	0.269	<0.001	0.324	<0.001	0.130	0.009	0.306	<0.001
CD28	0.412	<0.001	0.476	<0.001	0.334	0.334	0.206	<0.001
CD40	0.519	<0.001	0.553	<0.001	0.568	<0.001	0.463	<0.001
CD44	0.420	<0.001	0.441	<0.001	0.401	<0.001	0.576	<0.001
CD48	0.451	<0.001	0.589	<0.001	0.564	<0.001	0.511	<0.001
CD70	0.121	<0.001	0.106	0.005	0.114	0.023	−0.130	0.034
CD80	0.417	<0.001	0.612	<0.001	0.418	<0.001	0.314	<0.001
CD86	0.865	<0.001	0.816	<0.001	0.817	<0.001	0.858	<0.001
CTLA4	0.078	0.044	0.308	<0.001	−0.085	0.089	−0.084	0.171
HAVCR2	0.878	<0.001	0.827	<0.001	0.670	<0.001	0.796	<0.001
HHLA2	0.004	0.912	0.192	0.192	−0.200	<0.001	−0.102	0.095
ICOS	0.446	<0.001	0.369	<0.001	0.236	<0.001	0.153	0.012
ICOSLG	0.296	<0.001	0.543	<0.001	0.405	<0.001	0.254	<0.001
LAG3	0.099	0.009	0.201	<0.001	0.268	<0.001	0.015	0.810
LAIR1	0.865	<0.001	0.832	<0.001	0.842	<0.001	0.869	<0.001
LGALS9	0.764	<0.001	0.719	<0.001	0.798	<0.001	0.801	<0.001
NRP1	0.367	<0.001	0.375	<0.001	0.297	<0.001	0.366	<0.001
PDCD1	0.340	<0.001	0.131	<0.001	−0.110	0.028	−0.201	<0.001
PDCD1LG2	0.531	<0.001	0.616	<0.001	0.372	<0.001	0.370	<0.001
TMIGD2	0.491	<0.001	0.226	<0.001	0.026	0.610	0.006	0.925
TNFRSF18	0.122	0.001	0.048	0.206	0.260	<0.001	−0.091	0.139
TNFRSF25	0.100	0.010	−0.004	0.927	0.127	0.011	0.069	0.260
TNFRSF4	0.084	0.030	0.068	0.072	0.119	0.018	−0.168	0.006
TNFRSF8	0.248	<0.001	0.265	<0.001	0.200	<0.001	−0.051	0.405
TNFRSF9	0.255	<0.001	0.293	<0.001	0.139	0.005	−0.057	0.349
TNFSF14	0.353	<0.001	<0.001	<0.001	0.513	<0.001	0.234	<0.001
TNFSF15	0.457	<0.001	0.510	<0.001	0.337	<0.001	0.149	0.014
TNFSF18	0.146	<0.001	0.247	<0.001	0.039	0.441	−0.008	0.893
TNFSF9	0.009	0.808	0.220	<0.001	−0.030	0.548	−0.117	0.055
VTCN1	−0.058	0.130	0.126	<0.001	0.035	0.480	−0.186	0.002

### Identification and Validation of Spleen Tyrosine Kinase-Related Subtypes in Diffuse Glioma

**FIGURE 9:**
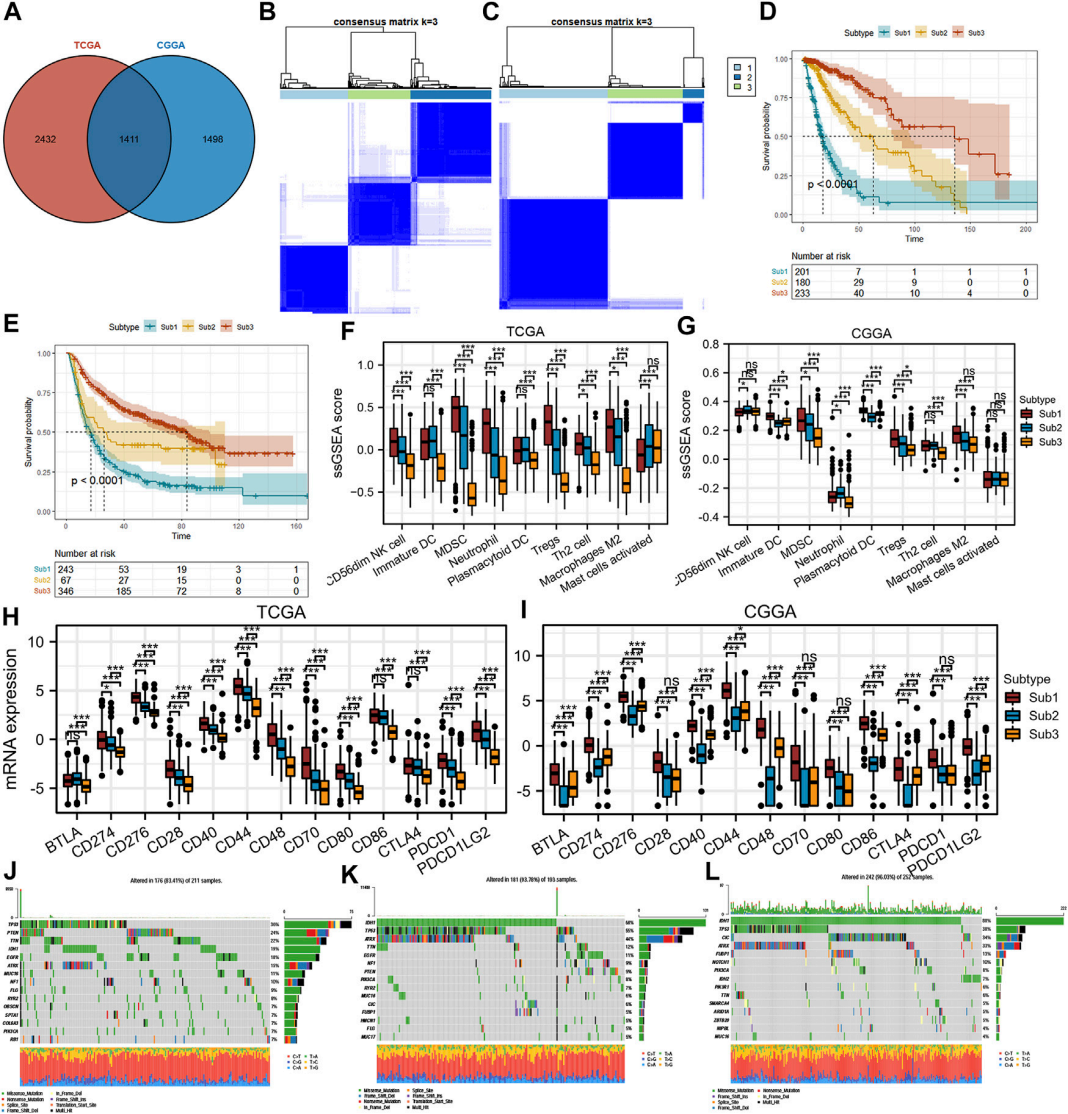
Differences in protumor immune cells, immune checkpoint markers, mutations, and the prognosis of SYK-associated subtypes in the TCGA and CGGA cohorts. **(A)** Identification of SYK-associated genes in the TCGA and CGGA cohorts. **(B–C)** Identification and validation of SYK-associated subtypes in the TCGA **(B)** and CGGA cohorts **(C)**. *p* values were calculated by the log-rank test, and *p* < 0.05 was considered significant. **(D–E)** The difference in prognosis between diffuse glioma with SYK-associated subtypes in the TCGA **(D)** and CGGA cohorts **(E)**. **(F–G)** The difference in protumor immune cells between diffuse glioma with SYK-associated subtypes in the TCGA **(F)** and CGGA cohorts **(G)**. **(H–I)** The difference in immune checkpoint markers between diffuse glioma with SYK-associated subtypes in the TCGA**(H)** and CGGA cohorts **(I)**. **(J–L)** Mutational profiles of SYK-associated subtypes (- no significance, *p < 0.05, and ***p < 0.001).

To screen patients with diffuse glioma suitable for blocking SYK, the SYK-related subtypes need to be identified. We defined 1,411 genes that were correlated with SYK expression (absolute Pearson r > 0.3, *p* < 0.001) by Pearson correlation analysis in the TCGA and CGGA cohorts (Figure 9A) (Supplementary Table S1). Based on the profiles of 1411 SYK-related genes, we performed NMF consensus clustering to identify three subtypes (Sub1, Sub2, and Sub3) in the TCGA cohort, which was validated in the CGGA cohort (Figures 9B, C). Importantly, diffuse glioma patients with subtype Sub1 (median survival, 17.3 months) had significantly shorter OS in the TCGA cohort, compared with those with subtype Sub2 (median survival, 63.0 months; HR = 0.31, 95% CI = 0.22–0.42, log-rank test *p* < 0.001) and Sub3 (median survival, 136.3 months; HR = 0.10, 95% CI = 0.07–0.16, log-rank test *p* < 0.001) (Figure 9D). Furthermore, similar prognostic difference was validated in the CGGA cohort, with diffuse glioma with subtype Sub1 (Sub1, median survival, 16.9 months) showing a significantly shorter OS than those with Sub2 (median survival, 26.0 months; HR = 0.60, 95% CI = 0.43–0.84, log-rank test *p* < 0.001) and Sub3 (median survival, 83.7 months; HR = 0.33, 95% CI = 0.27–0.41, log-rank test *p* < 0.001) (Figure 9E).

Next, we explored the correlations between the SYK-associated subtype and protumor immune cells (CD56dim NK cells, immature DCs, MDSCs, neutrophils, plasmacytoid DCs, Tregs, Th2 cells, activated mast cells, and M2 macrophages). Significant differences were observed between Sub1 and the other two subtypes, with a higher abundance of seven immune cell populations (immature DCs, MDSCs, neutrophils, plasmacytoid DCs, Tregs, Th2 cells, and M2 macrophages) for Sub1 compared with Sub2 or Sub3 in the TCGA cohort and CGGA cohort (Figures 9F, G). We speculated diffuse glioma with Sub1 was accompanied by an immunosuppressive tumor microenvironment. However, glioma with Sub1 did not exhibit a higher abundance of activated mast cells and CD56dim NK cells than Sub2 and Sub3 (Figures 9F, G).

We further investigated the association between subtypes and the expression of 13 potentially targetable immune checkpoint genes, such as PD-L1, PDCD1, and PD-L2, and the results showed that Sub1 exhibited higher expression of 13 immune checkpoint genes than Sub2 and Sub3 in the TCGA cohort (*p* < 0.05, Figure 9H). A similar difference was observed, with Sub1 showing significantly higher expression of immune checkpoint genes than that for Sub2 and Sub3 in the CGGA cohort (*p* < 0.05, Figure 9I). In addition, a waterfall plot was used to show the top 15 most frequently mutated genes in each subtype (Figures 9J–L). The mutation rates of IDH1 and ATRX were significantly lower in Sub1 (IDH1,18% and ATRX, 15%) than Sub2 (IDH1, 68% and ATRX, 44%) and Sub3 (IDH1, 88% and ATRX, 33%), while the mutation rates of PTEN, TTN and EGFR were significantly higher in Sub1 (PTEN, 24%, TTN, 22% and EGFR, 18%) than in Sub2 (PTEN, 9%, TTN, <5% and EGFR, 11%) and Sub3 (PTEN, <4%, TTN, 6% and EGFR, <4%) (Figures 9J–L). In conclusion, targeting SYK alone or in combination with other immune checkpoint inhibitors is more likely to show a benefit in diffuse glioma with Sub1 by improving the immunosuppressive tumor microenvironment and thus the patient prognosis.

## Discussion

Diffuse glioma is the most frequent primary brain tumor and it is universally fatal and recurrent, despite multimodal treatments. Therefore, it is necessary to develop new approaches to treating glioma. Diffuse glioma is characterized by a highly immunosuppressive and heterogeneous tumor microenvironment that is considered a key regulator of malignant progression (16**–**17, (Cancer Genome Atlas Research Network, 2008)). The discovery of the lymphatic system of the central nervous system may provide theoretical support for neuroimmunology (Louveau et al., 2015). Recently, different immunotherapeutic approaches have proven to be successful in treating many malignant cancers (Del Paggio, 2018). The discovery of new molecular targets for immunotherapy will be beneficial in improving the prognosis of glioma patients.

SYK has been reported in the literature to play a role in promoting and inhibiting tumors (Ghotra et al., 2015; Krisenko and Geahlen, 2015; Yu et al., 2018; Liu et al., 2020). In this study, we also found SYK was heterogeneously expressed in different normal tissues and cancer cells (*p* < 0.05, Supplementary Figures S1A, B), and was significantly differently up-expressed and down-expressed in the multiple tumors (*p* < 0.05, Supplementary Figures S1C, D), which was consistent with SYK’s dual role as a tumor promoter and tumor suppressor. Importantly, high expression of SYK was frequently observed in the malignant phenotype in the four cohorts, which was further validated by qPCR and IHC (*p* < 0.05, Figures 1, 2). Furthermore, SYK was found to be an unfavorable prognostic marker, independent of other clinicopathologic factors (*p* < 0.05, Figures 3G, H). SYK has been shown to promote the proliferation and migration of glioma cells and recruit macrophages into the tumor microenvironment (Moncayo et al., 2018; Yan et al., 2021). Therefore, SYK could play a protumorigenic role in diffuse gliomas. However, the potential of SYK as an immunotherapeutic target still needs to be explored in diffuse glioma.

Accumulating evidence has shown that gliomas are brain tumors characterized by an immunosuppressive microenvironment, which limits the efficacy of cancer therapy (Yang et al., 2020; van Hooren et al., 2021). In addition, SYK was found to contribute to modifying the tumor microenvironment in some tumors (Herman et al., 2011; Moncayo et al., 2018). Inhibition of SYK could affect the tumor microenvironment by regulating macrophage polarization and by partially blocking monocyte and B-cell infiltration (Joshi et al., 2014; Moncayo et al., 2018). A sequence of immune infiltration analyses showed abnormally expressed SYK could contribute to the immunosuppressive tumor microenvironment, caused by immunosuppressive cells (CD56dim NK cells, immature DCs, MDSCs, neutrophils, plasmacytoid DCs, Tregs, Th2 cells, and M2 macrophages). Because of the immunosuppressive microenvironment caused by immunosuppressive cells, the association between SYK expression and pro-tumor immune cells (CD56dim NK cells, immature DCs, MDSCs, neutrophils, plasmacytoid DCs, Tregs, Th2 cells, and M2 macrophages) should be investigated. We found that diffuse gliomas with high SYK expression had a higher abundance of immunosuppressive cells than those with low SYK expression (Figures 5, 6). The previous literature reported that SYK was responsible for immune cell infiltration into the tumor microenvironment (Moncayo et al., 2018; Yan et al., 2021). SYK is expressed by both glioma tumor cells and leukocytes (Moncayo et al., 2018). Gliomas are highly infiltrated by bone marrow-derived macrophages and brain-resident microglia, which promote tumor growth and suppress the immune response (Hambardzumyan et al., 2016). The abnormal expression of SYK in tumor cells or leukocytes may activate several signaling pathways to promote the release of some chemokines (Kodar et al., 2017; Li et al., 2020; Itoh et al., 2021). We also found that tumors with high SYK expression were enriched in NF-kappaB signaling **(** Figure 4F). However, the association between SYK and M2 macrophage infiltration remains unclear in diffuse glioma. We identified 18 cell clusters by the single-cell data sequencing analysis (Figure 7A) and found the expression pattern of SYK in the identified cell clusters was most similar to that of the M2 macrophage markers CD163 and VSIG4, which was validated by correlation analysis (Figures 7B–H). The literature indicates that SYK plays an important role in differentially modulating M1-like and M2-like macrophage phenotypes (Joshi et al., 2014; Kodar et al., 2017). SYK kinase has been extensively studied in adaptive immune responses, but its role in macrophage-mediated innate immune responses remains unclear (Yi et al., 2014). These results indicated that aberrant expression of SYK was involved in shaping the immunosuppressive microenvironment in diffuse glioma. However, SYK expression had an inverse correlation with the infiltrating levels of CD8^+^ T cells between LGG and GBM (*p* < 0.05, Figure 5). Generally, CD8^+^ cells play a role in tumor suppression. Many types of CD8^+^ are included in the tumor microenvironment, and each type of cell has different tumor-suppressive effects and intensities. Using TIMER analysis, we can only estimate the total number of CD8^+^ cells of all types. GBM is obviously a more immunosuppressive tumor, lacking more CD8^+^ cells than LGG. The infiltration of T cells, especially CD8^+^ T cells, into the tumor microenvironment correlates with a better prognosis in brain cancer (Vesely et al., 2011). However, even when tumor-specific CD8^+^ T-cell responses are observed, they rarely provide protective immunity, as tumors often evade immune surveillance by dampening T-cell effector and memory functions (Bindea et al., 2013). Moreover, SYK might recruit more immunosuppressive immune cells, such as M2 macrophages. The state of the immune microenvironment is determined by the interaction between immunosuppressive and immunoactivated cells.

In addition, SYK expression was closely associated with most chemokines, such as CXCL16 and CXCL12 (Figure 8B). The previous literature showed that the activation of the SYK/NF-κB signaling pathway could accelerate M2 polarization and tumor progression (Li et al., 2020). Tumor-associated macrophages and neutrophils are associated with an unfavorable prognosis in diffuse glioma patients (Zhang et al., 2017; Wei et al., 2018) and facilitate the shape of the immunosuppressive tumor microenvironment. Activation of macrophages and neutrophils can produce tumor necrosis factor (TNF) and induce pro-inflammatory cytokines, such as IL-1, and promote tumorigenesis (Balkwill, 2009). A large proportion of the tumor microenvironment consists of an inflammatory infiltrate predominated by microglia and macrophages, which are thought to be subverted by glioblastoma cells for tumor growth (Poon et al., 2017). Thus, glioblastoma-associated microglia and macrophages are logical therapeutic targets. Their emerging roles in glioblastoma progression are reflected in the burgeoning research into therapeutics directed at their modification or elimination. Therefore, inhibition of SYK might be a treatment option for modulating the microenvironment and inhibiting tumor progression.

Currently, many patients with advanced melanoma and advanced melanoma benefit greatly from immunotherapy checkpoint-targeted immunotherapy, which has achieved remarkable success in some tumors (Topalian et al., 2012; Wolchok et al., 2013; Rizvi et al., 2015). Combination treatments for melanoma were shown to be more effective than monotherapy treatments (Larkin et al., 2015). Pembrolizumab and nivolumab are well tolerated; however, progression-free survival and immune analyses have indicated that anti-PD-1 monotherapy is insufficient to produce an antitumor response in the majority of patients with GBM due to high levels of CD68^+^ cells and few T cells within the tumor microenvironment (Chamberlain and Kim, 2017). In addition, the efficacy of immune checkpoint inhibitors has been unsatisfactory in GBM (Zhao et al., 2019). Therefore, novel molecules that can collaborate with immune checkpoint molecular blockade to improve the therapeutic efficacy need to be identified.

Here, we found that SYK might exert synergistic effects with multiple immune checkpoint molecules and immunogenic cell death modulators across cancers (Figure 8A). Importantly, in the four cohorts, SYK expression was closely associated with several immune checkpoint molecules, such as CD276, CD44, CD80, CD86, and PD-L2 (Table 1). Clinical trials or clinical situations have confirmed the amazing effect of blocking immune checkpoints in multiple tumors (Lim and Soo, 2016; Ribas and Wolchok, 2018; Feng et al., 2019). We also found that SYK expression had a close association with TMB, the immunotherapy response, in pancancer (Figure 8). However, we also found that SYK expression was positively correlated with some immune coinhibitory molecules, such as PD-L1, and some immune costimulatory molecules (such as CD40 and ICOS) (Table 1). It is generally thought that PD-L1 functions within the tumor bed, where cell-surface PD-L1 directly interacts with PD-1 on the surface of tumor-infiltrating lymphocytes (TILs) (Poggio et al., 2019). SYK is expressed by glioma cells (Moncayo et al., 2018). Therefore, we speculated that both SYK and PD-L1 were expressed by glioma cells and that their expression levels were highly correlated. However, some immune costimulatory molecules, such as CD40, are also positively correlated with the expression of SYK, which is worth exploring. In B cells, SYK is essential, as its deficiency disrupts signaling from the pre‒B-cell receptor complex, leading to the complete depletion of mature B cells (Turner et al., 1995). SYK expression is positively correlated with CD40 expression. SYK can be found in the cytoplasm and the nucleus due to an unconventional shuttling sequence (Zhou et al., 2006). SYK has been found to act as a transcription factor. We hypothesized that SYK might transcriptionally regulate the mRNA expression of some immune coinhibitory molecules, such as PD-L1, and some immune costimulatory molecules, such as CD40. Therefore, the expression of SYK is positively correlated with that of some immune coinhibitory and costimulatory molecules. Importantly, when we explored PD-L1 and SYK using the glioma tissue microarrays, a positive correlation was observed (Pearson test, *p* < 0.05, Figures 8D, E). To screen patients with diffuse glioma suitable for blocking SYK, SYK-related subtypes were identified. Diffuse glioma with Sub1 exhibited a worse prognosis, an immunosuppressive tumor microenvironment, significantly higher expression of immune checkpoint genes, and distinct mutation characteristics (Figure 9), which suggested diffuse glioma with Sub1 is more likely to benefit from blocking SYK alone or in combination with other immune checkpoint inhibitors. However, the synergistic effect of simultaneously targeting SYK and other immunoassay molecular blockages has not been tested so far.

Although we found that SYK is associated with a malignant phenotype and contributes to shaping the immunosuppressive tumor microenvironment in diffuse glioma, this study still has the following limitations. First, a few contradictory results need to be validated by solid experimental data. Second, the prognostic value of SYK in gliomas has not been confirmed in the real world. Finally, more functional experiments are needed to validate the role of SYK in the glioma microenvironment, especially the immune microenvironment. Importantly, the potential mechanism by which SYK regulates the polarization of M2 macrophages or recruits M2 macrophage infiltration needs further exploration.

In conclusion, SYK is abnormally expressed in different tumors. Importantly, diffuse glioma with high SYK expression was accompanied by a malignant phenotype and had a poor prognosis. Functionally, SYK might be involved in immune-associated processes and shape the immunosuppressive tumor microenvironment. SYK is a promising biomarker and immunotherapeutic target for diffuse glioma.

## Data Availability

Publicly available datasets were analyzed in this study. This data can be found here: Chinese Glioma Genome Atlas (CGGA, http://www.cgga.org.cn/), The Cancer Genome Atlas (TCGA, https://tcga-data.nci.nih.gov/tcga/) databases, GSE16011 database (*n* = 276), and the Rembrandt database (*n* = 471) (https://www.ncbi.nlm.nih.gov/geo).

## Supplementary Material

Supplemetary Figure S1 The expression profiles of SYK in multiple tissues, cells, and tumors. **(A)** The expression profile of SYK in 31 types of normal tissues; **(B)** The expression profile of SYK in 21 types of tumor cells; **(C)** Differential expression of SYK between tumor and normal tissues from the TCGA dataset; **(D)** SYK expression in 27 cancer types from the GTEx and TCGA databases (- no significance, **P* < 0.05, ***P* < 0.01, and ****P* < 0.001).

Supplemetary Figure S2 The association between SYK expression and the expression levels of immune checkpoint-associated molecules in pancancer.

Supplemetary Figure S3 Flowchart of this study.
