# Supplementary figures and images for: SYK Is Associated With Malignant Phenotype and Immune Checkpoints in Diffuse Glioma
Source: Front Genet. 2022 Jul 15;13:899883. doi: 10.3389/fgene.2022.899883 (PMC9334658; doi:10.3389/fgene.2022.899883)

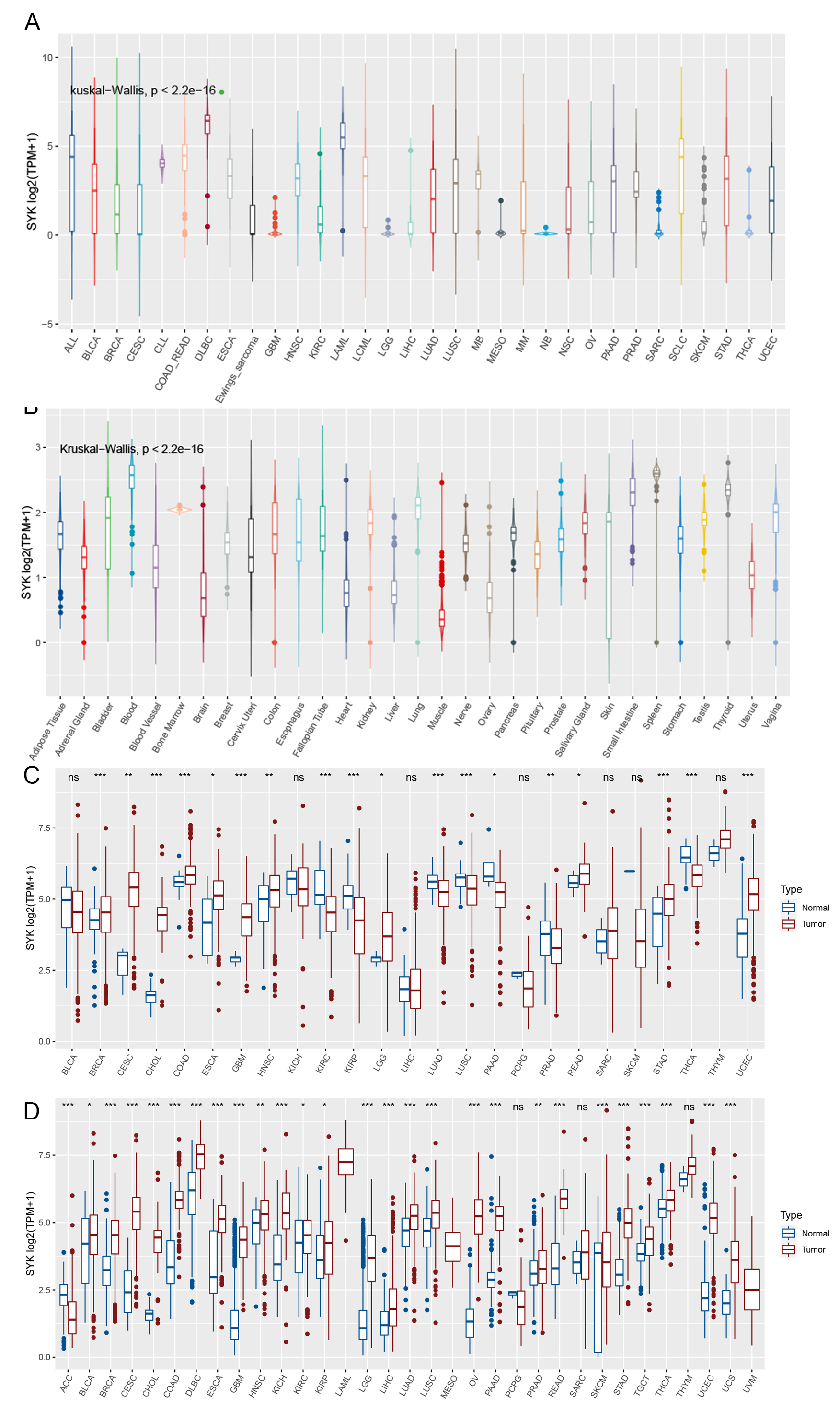

Supplement: Supplementary file 1 [file DataSheet1.zip › supplementary file/FS1.tif]

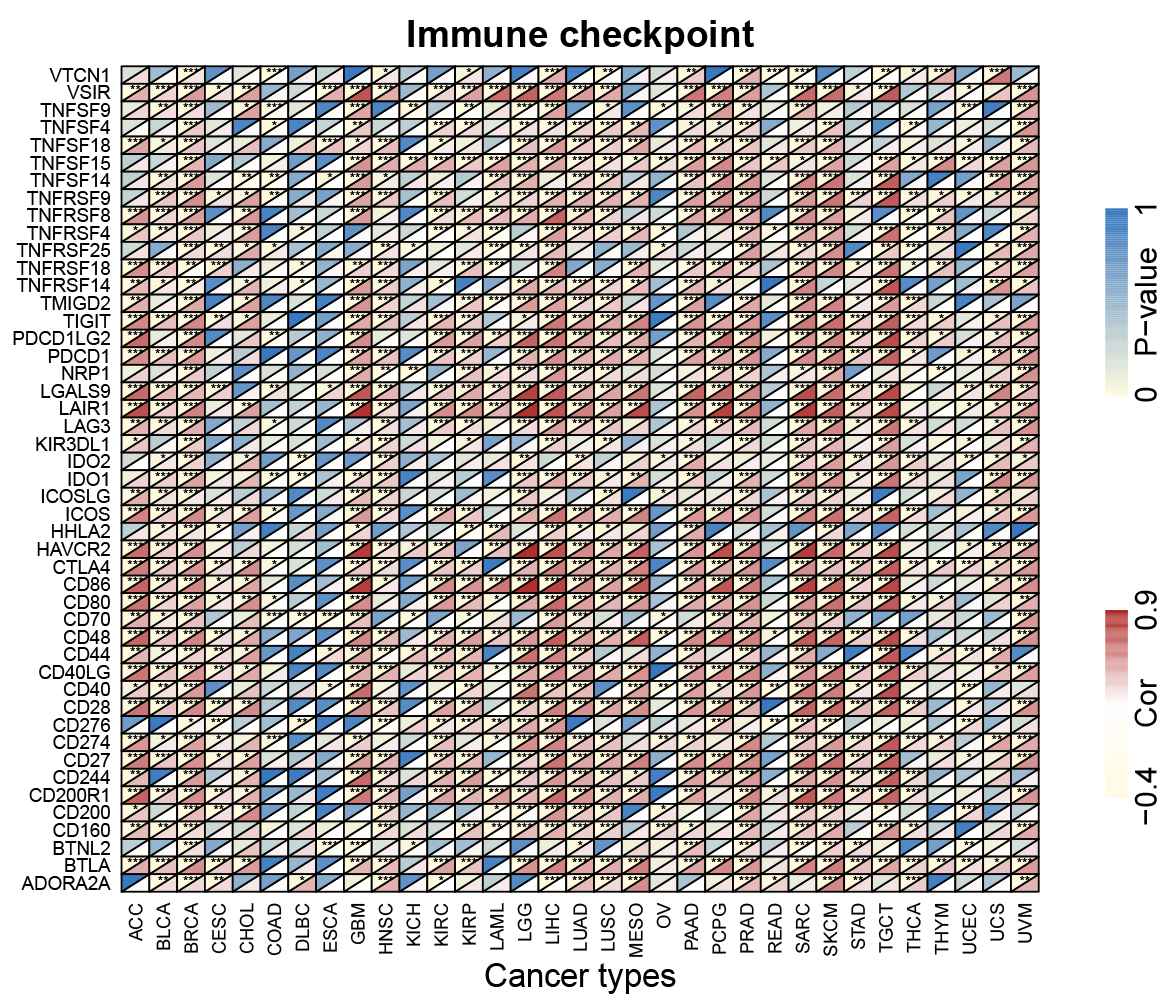

Supplement: Supplementary file 1 [file DataSheet1.zip › supplementary file/FS2.tif]

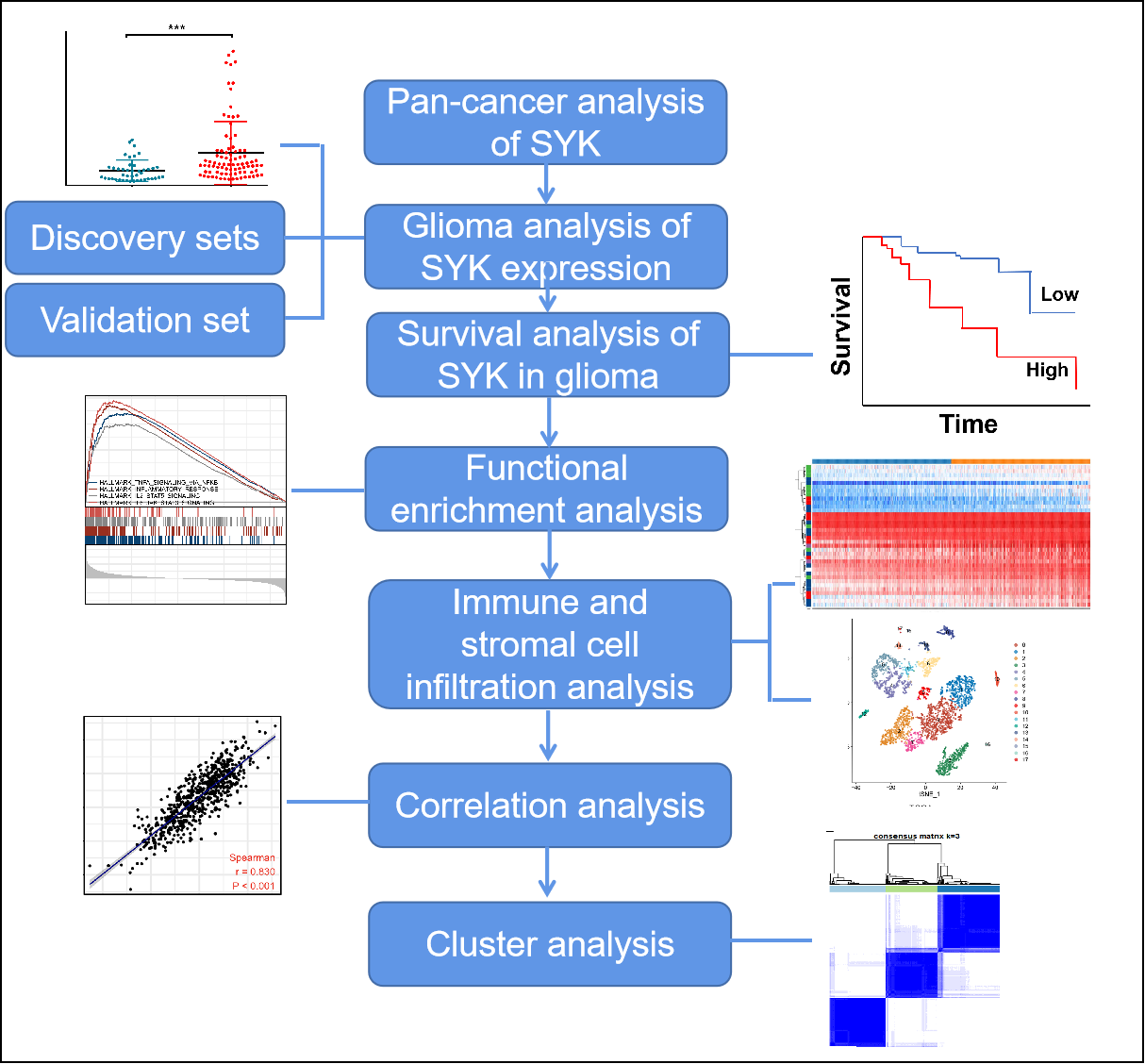

Supplement: Supplementary file 1 [file DataSheet1.zip › supplementary file/图片1.png]
